# A Longitudinal Study of Aggression in People with Autism and Other Neurodevelopmental Disabilities

**DOI:** 10.1007/s10803-024-06559-0

**Published:** 2024-09-27

**Authors:** Dena Gohari, Hillary Schiltz, Catherine Lord

**Affiliations:** Semel Institute for Neuroscience and Human Behavior, University of California, Los Angeles, 760 Westwood Plaza, Los Angeles, CA 90024 USA

**Keywords:** Aggression, Autism, Longitudinal, Neurodevelopmental disabilities

## Abstract

Aggression is common in autism and neurodevelopmental disorders, but longitudinal research on aggression is lacking. We longitudinally tracked aggression in 254 individuals from toddlerhood to emerging adulthood. Our sample included participants with a range of cognitive abilities, with 39.9% classified as more-cognitively-abled (MCA; IQ ≥ 70) and 60.1% as less-cognitively-abled (LCA; IQ < 70). Aggression Composite scores were derived from data from the autism diagnostic observation schedule, autism diagnostic interview-revised, and child behavior checklist at ages 2, 9, and 18. Fifty-four percent, 69%, and 42% of the sample showed aggression in toddlerhood, school age, and emerging adulthood, respectively. LCA individuals had higher rates of aggression in school age (80%) and emerging adulthood (58%) compared to MCA individuals (48 and 22%, respectively). Longitudinal aggression profiles revealed distinct patterns of change over time: 31% displayed persistent aggression, 25% increased, 23% decreased, and 13% never displayed aggression. Higher autism symptoms, lower VIQ, NVIQ, and less-developed adaptive skills correlated with more aggression cross-sectionally. Nonverbal IQ and repetitive behaviors related to aggression longitudinally: people in decreasing or absent profiles had higher NVIQ and fewer RRBs than those with persistent or increasing profiles. Participants with aggression at 9 were four times likelier to exhibit aggression at 18. Aggression is common in autism and NDDs, peaking around age 9, and declining in emerging adulthood. Patterns of change varied widely, with evidence that higher NVIQ and fewer RRBs may be protective. Findings have implications for clinical practices, highlighting important developmental periods and high-risk subgroups.

## Introduction

People with autism spectrum disorder (ASD) or other neurodevelopmental disabilities (NDDs) often experience higher rates of behaviors that others may find challenging, such as aggression (De Giacomo et al., 2016; Hartley et al., 2008; Quetsch et al., 2023). Aggression—behavior that is threatening or likely to cause harm (Fitzpatrick et al., 2016)—can be physical, verbal, or a combination of modalities towards either caregivers or others (Hartley et al., 2008; Matson & Cervantes, 2014; Mazurek et al., 2013). Aggression is often reported as a primary concern for autistic children by parents and teachers (Azad & Mandell, 2016). In some cases, aggression can catalyze a myriad of ripple effects, including familial estrangement, social isolation, refusal of services, property destruction, and caregiver stress (Neece et al., 2012). As such, aggression can play a central role in the lives of individuals with autism and other NDDs, as well as their families. Existing research on aggression in autism and NDDs, however, has primarily focused on childhood cross-sectionally using parent-reported instruments. Multi-method assessment of aggression over time, especially beyond childhood, remains limited and is therefore the focus of the current study.

### Rates of Aggression in Autism and NDDs Across Development

Although estimates vary across studies, rates of aggression in autistic individuals are typically higher than in non-autistic samples without other developmental delay (e.g., general population studies; Quetsch et al., 2023; Sivathasan et al., 2023) and other psychiatric conditions (Blanchard et al., 2021; Matson et al., 2009; Mazurek et al., 2013; Pugliese et al., 2013; Taylor et al., 2011) and may vary in accordance with developmental level (Laverty et al., 2023). Average prevalence estimates of autistic individuals’ current aggression towards caregivers and non-caregivers during childhood are approximately ~ 60 and ~ 30%, respectively (Kanne & Mazurek, 2010; Mazurek et al., 2013). To date however, few studies have examined the prevalence of aggression beyond childhood, with findings of these studies limited by sample characteristics. More specifically, much of the current literature on aggression has focused on those with intellectual disability (ID), while little work has included autistic people across a range of cognitive abilities. In one of few studies on adults, Matson and Rivet (2007) found that between 15 and 18% of adults with autism and co-occurring ID exhibited aggression based on a clinician-rated questionnaire completed by doctoral students who had known participants for at least six months. It remains unclear how often aggression persists from childhood through adolescence or adulthood among autistic people and to what extent adults with aggression demonstrated these behaviors as children. Such questions may only be answered with longitudinal data.

The recent work of Laverty and colleagues (2023) is the only study to our knowledge that examines longitudinal trajectories of aggression among autistic individuals with and without ID. Two hundred and twenty-nine individuals with autism were followed over approximately ten years at ages 12, 15, and 24, with a final sample of 54 participants at the last time point. Controlling for attrition, rates of aggression estimated from a parent-report questionnaire—the challenging behavior questionnaire (Hyman et al., 2002)—were found to attenuate from 67% at age 12 to rates of approximately 30% at age 24. Aggression was found to persist over time for 30% of their sample, substantially higher than estimates of about 10% reported among neurotypical adults (Broidy et al., 2003; Laverty et al., 2023). The current study builds upon these findings by extending the timespan to earlier and later developmental periods (i.e., age 2 and age 25, years respectively) and by employing a multimethod approach to assess aggression.

Research on aggression in autism and other NDDs needs to be interpreted according to study methodology, particularly method of aggression measurement. Aggression has often been assessed using a single parent-reported dichotomous questionnaire item (e.g., parents’ responses to one yes-or-no item; Cooper et al., 2009; Kanne & Mazurek, 2010) or the two aggression items on the autism diagnostic interview-revised (ADI-R; 81 & 82; Kanne & Mazurek, 2010). However, complexities of aggression may not be fully captured by such metrics alone. Aggression can be verbal and/or physical, occur at varying levels of severity, and may be directed towards caregivers or non-caregivers. Context may also play a role in aggression (e.g., home vs. public spaces vs. clinic settings; Farmer et al., 2016; Pouw et al., 2013). As such, multimethod approaches that include a combination of observational, interview, and questionnaire-based tools across multiple perspectives and contexts may help ensure more accurate estimation of aggression rates. This approach has not been used in this area of study to date.

### Correlates of Aggression

Some people may be more likely to display aggression based on a combination of factors. In cross-sectional studies, rates of aggression in autistic individuals are correlated with autism-specific behaviors, cognitive abilities, and adaptive functioning. Aggression has been significantly linked to greater autism symptom severity, including sensory difficulties and the greater presence of restricted and repetitive behaviors (RRBs; Fitzpatrick et al., 2016; Hartley et al., 2008; Mazurek et al., 2013; Sullivan et al., 2019; van den Boogert et al., 2021). Additionally, other co-occurring externalizing behaviors such as self-injury, hyperactivity, and irritability, have been associated with aggression in autism (Baweja et al., 2023; Fitzpatrick et al., 2016; Hartley et al., 2008; Laverty et al., 2023; Mazurek et al., 2013).

Some research found low IQ, both verbal and nonverbal, and ID to relate to a greater presence of aggression (Hartley et al., 2008; Mazurek et al., 2013), but other studies have not, likely due to differences in ranges of ID in included participants (i.e., Baweja et al., 2023; Brown et al., 2019; Esteves et al., 2021). Results become even more complex when considering multiple factors simultaneously. While several studies report that co-occurring autism and ID result in higher rates of aggression relative to ID alone (Mazurek et al., 2013; McClintock et al., 2003; Nicholls et al., 2019), others found autism diagnosis to attenuate aggression severity in intellectually disabled individuals (Farmer et al., 2016). Taken together, these studies highlight how multiple factors, including autism symptoms, cognitive functioning, co-occurring externalizing and internalizing symptoms, and adaptive ability, may place an autistic person at greater risk for aggressions. The present paper uses a longitudinal approach to consider the role of these factors, namely autism symptoms and cognitive ability, in both chronic and acute levels of aggression across development.

### Study Aims

The present study leverages a rich longitudinal sample of people with autism or other non-spectrum delays with a range of cognitive abilities followed from toddlerhood through emerging adulthood to address four central aims: (1) to characterize rates of aggression at various developmental time points (toddlerhood: age 2; school-age: age 9; and emerging adulthood: age 18) using a composite score based on clinician observation, clinical interview, and parent-reported questionnaire; (2) determine patterns of changes in aggression over time; (3) identify factors related to various trajectories of aggression over time; and (4) examine whether the presence of early aggression is predictive of aggression later in life. Because literature on longitudinal trajectories of aggression is quite limited, our study does not have specific hypotheses for each of these aims; however, we would expect the overall trend of aggression to decline over time based on cross sectional data and the work of Laverty et al (2023).

## Method

### Participants

The present study reports data from a sample of 254 participants enrolled in an ongoing longitudinal study of people with autism spectrum disorder or other NDDs. Participants were initially recruited from referrals to three autism program sites (North Carolina, Illinois, and Michigan), though not all received autism diagnoses. Previous investigations have yielded similar patterns in the autism and non-autism DD participants (Lord et al., 2020); thus, all participants were retained in analyses to provide comparison groups and maximize sample size. We also examined potential differences in aggression between the two diagnostic (autism vs. other NDD) groups to further justify combining our samples (see preliminary analyses below). At the start of the study in the early 1990s, children recruited from North Carolina and Illinois were under the age of 3, and children recruited from Michigan all had early diagnoses but joined the study when they were approximately 9 years old. A majority of the sample is White (82.3%), and male (77.6%), with maternal education of a college degree or more (62.3%). Participants were classified as either more-cognitively-abled (MCA; 39.9%) or less-cognitively-abled (LCA; 60.1%) based on an age 9 IQ cut-off of 70 consistent with prior work (Lord et al., 2020). See Table 1 for additional sample demographics.

**Table 1:** Participant demographics

	ASD (N = 196)	DD (N = 58)	Chi-squared	*p*
Race			0.134	0.715
White/Caucasian	135	40		
Person of color	61	16		
Ethnicity			1.02	0.314
Hispanic	5	3		
Not hispanic	188	54		
Site			**37.3**	** < 0.001**
North Carolina	105	27		
Chicago	74	7		
Michigan	17	23		
Maternal education			2.78	0.095
< 4 years of higher education	89	27		
> 4 years of higher education	89	15		
Sex			**23.2**	** < 0.001**
Male	170	33		
Female	26	24		

Bold values indicate significant differences at the .05 level

### Procedure

The longitudinal study, first launched in 1992 and still ongoing, was approved by Institutional Review Boards at relevant universities throughout the study. Caregivers and participants themselves provided informed consent (when possible) or assent for their participation in the study. Both in-person assessments (including diagnostic and cognitive testing) as well as bi-annual questionnaire packets completed via mail or online have been employed. In-person assessments occurred throughout the longitudinal study (ages 2, 3, 5, 9, 18, 21, 26, and 30). Clinicians conducting the in-person assessments were research reliable on the relevant measures and masked to participants’ previous assessment results. The research team presented all recently collected information to a panel of experienced clinicians who also had access to previous data and made consensus diagnoses of autism and other conditions. All assessments were provided free of charge and included feedback on testing results. For the purposes of the current analyses, data were selected to focus on toddlerhood (age 2), school-age (age 9), emerging adulthood (age 18), and when available, young adulthood (age 25). Power analyses conducted in G*Power determined the sample is sufficiently powered to detect large (0.4; *n* = 90) and medium (0.25; *n* = 216) effects but may be too small to detect small effects.

### Measures

#### Aggression

Consistent with a multimethod approach, three measures were used to capture aggression at three time points. These consisted of an observational code (ADOS; mean ages 2.44, 9.57, and 19.0), a clinician-administered parent interview (ADI; mean ages 2.41, 9.36, and 19.04 years), and a parent-report questionnaire (CBCL; mean ages 9.39, 17.53, and 25.44 years), described below. Sample sizes for each aggression measure are presented in Table 2. Aggression measures were combined in the current analyses to generate a binary Aggression Composite at each time point such that “1” on the composite indicates elevated aggression on at least one aggression measure and “0” on the composite indicates no evidence of aggression on any of the aggression measures.

**Table 2:** Descriptives and correlations among aggression measures

Variable	*n*	Med/mean (SD)	Min	Max	1	2	3	4	5	6	7	8	9	10	11
1. ADOS aggression 2	210	0	0	2	–										
2. ADOS aggression 9	163	0	0	2	0.09	–									
3. ADOS aggression 18	98	0	0	3	0.12	0.12	–								
4. ADI aggression 2 (TANT)	198	0	0	3	0.03	− 0.16*	− 0.14	–							
5. ADI aggression 9 (F)	160	0	0	3	0.10	0.21**	0.10	0.03	–						
6. ADI aggression 18 (F)	116	0	0	3	0.00	0.03	0.13	− 0.04	0.28**	–					
7. ADI aggression 9 (O)	160	0	0	3	0.07	0.17*	0.16	0.01	0.65**	0.25**	–				
8. ADI aggression 18 (O)	116	0	0	3	− 0.005	− 0.09	0.10	− 0.07	0.25**	0.63**	0.30**	–			
9. CBCL aggression 9	113	55.6 (8.9)	50	97	− 0.03	0.09	0.06	0.10	0.44**	0.25*	0.32**	0.12	–		
10. CBCL aggression 18	81	54.9 (6.3)	50	70	− 0.14	− 0.08	− 0.06	0.00	0.21	0.39**	0.36**	0.46**	0.28*	–	
11. CBCL aggression 25	89	53.8 (4.5)	50	66	− 0.14	0.01	− 0.14	0.24	0.14	0.34	− 0.03	0.21	0.49**	0.54**	–

For ordinal measures of aggression, (variables 1–8), medians were used

*ADOS* autism diagnostic observation schedule, *ADI* autism diagnostic interview revised, *TANT* tantrums, *F* aggression towards family, *O* aggression towards others, *CBCL* child behavior checklist, *2* 2 years old, *9* 9 years old, *18*  18 years old

##### Autism Diagnostic Observation Schedule (ADOS) E2 Code

The ADOS is a semi-structured standardized observational assessment of a participant’s behavior including communication, reciprocal social interaction, imagination/creativity, and stereotyped behaviors /RRBs, as well as other potentially interfering behaviors (e.g., aggression; Lord et al., 2001, 2012). Module selection is contingent on participants’ language level and chronological age. Including the toddler module, seven modules exist, including two adapted modules for less verbal adults. At age 2, participants received an earlier iteration of what is now the Toddler Module of the ADOS (formerly known as the PL-ADOS); modules at later ages varied according to the participants’ ages and language levels. The PL-ADOS (*n* = 212) and Module 1 (*n* = 42) were administered to participants at the first time point. Modules 1 (*n* = 110), 2 (*n* = 51) and 3 (*n* = 93) were administered at the second time point, and Modules 1 (*n* = 129), 2 (*n* = 5), 3 (*n* = 9), 4 (*n* = 72), and adapted Modules 1 and 2 (*n* = 39) were administered during the third time point.

The ADOS E2 code (ADOS-aggression) was used to measure aggression and tantrums in the current study. Scores range from 0 to 3, with higher scores reflecting greater frequency and/or intensity of aggression exhibited by the participant during the approximately one-hour duration of the assessment. For example, on Module 3, a score of 0 is indicative of “Not disruptive, destructive, negative, or aggressive during the ADOS-2 assessment,” while a score of 3 indicates “Shows marked or repeated temper tantrums or significant aggression (e.g., throwing things, hitting, or biting others). Screaming or yelling is included here.”

##### Autism Diagnostic Interview—Revised (ADI-R) Items 81 & 82

The ADI, and revised version ADI-R (noted throughout as “ADI”), are standardized semi-structured diagnostic interviews administered to a caregiver (Lord et al., 1993). A version of the original ADI modified to be used with children under age 5 was used between 1989 and 1994, and the revised ADI was used from 1994 onward; the aggression items did not change significantly between versions. Two items were used to capture aggression: aggression towards caregivers or family members (ADI-aggression-F; item 81) and aggression towards other people (ADI-aggression-O; item 82). Similar to the earlier version of the ADOS, the aggression item in the toddler/preschool version of the ADI probed for tantrum behaviors (e.g., 0 = “none or only rare and/or minor tantrums that parent has dealt without concern; 1 = occasional tantrums (not more than one a month)”; 2 = “frequent tantrums (more than 1 a month, but not once a week)”; 3 = “tantrums frequent (once or more a week) or so severe in terms of possible danger to child or others as to disrupt family functioning or limit activities”). Items in the revised version are also scored on a scale from 0 to 3, with higher values reflecting greater severity of aggression (i.e., in the most severe cases, with the use of implements). For example, a score of 1 indicates “mild aggressiveness only,” which includes “threatening without physical contact; or behavior that might represent just unduly rough play or momentary, provoked lashing out.”

##### Child Behavior Checklist (CBCL) Aggression Subscale

The CBCL (Achenbach & Edelbrock, 1991) is a standardized, age-normed parent-report questionnaire that assesses children and adolescents’ (age 6–18) behavioral-emotional challenges and strengths on a three-point Likert rating (0 = not true; 1 = somewhat/sometimes true; 2 = very/often true). In the current paper, the aggression subscale (consisting of 32 items) was used in analyses and will be referred to as “CBCL-Aggression.” Item responses were summed, and T-scores were generated from raw scores, with higher scores indicative of more severe aggression.

#### Factors Related to Aggression

Measures of autism features, cognitive functioning, and adaptive skills were used to examine relationships between these particular factors and aggression (cross-sectionally and longitudinally).

#### Autism Features

##### ADOS Calibrated Symptom Severity (CSS)

The ADOS CSS Social Affect (SA) and RRB scores were used respectively to indicate the degree to which participants exhibited challenges in social-communicative and RRB domains throughout the ADOS observation (described above). These behaviors are coded on a scale of 0–3, with 0 representing the absence of unusual behaviors and 3 indicating substantial evidence for autistic symptomatology. The total sum (with 3’s converted to 2’s) is converted to a calibrated severity score quantifying level of social communication challenges controlling for age and language level. A similar calibrated severity score is available for RRBs, using the sum of individual items including unusual sensory interests, hand and finger mannerisms, and unusually repetitive behaviors, coded on a scale of 0–3, where 0 indicates no evidence of RRBs and 3 indicates substantial evidence of RRBs. In the current analyses, CSS SA and RRB scores from the ADOS were employed in analyses examining the relationship between autism features and aggression.

##### Autism Diagnostic Interview—Revised (ADI-R) Domain Scores

Total scores from the three domains defining autism were used, including social interaction, qualitative abnormalities in communications, and RRBs. As indicated by the ADI algorithm, select items are summed to create total summary scores for each domain. The social interaction domain consists of four subdomains with a total of 15 items scored on a scale from 0 to 3. Higher scores indicate more substantial evidence of behaviors related to challenges in peer relationships, shared enjoyment, and social-emotional reciprocity. Qualitative abnormalities in communication consist of seven items measuring gestures and make-believe or social imitative play. Only nonverbal communication algorithm items were used for all participants for the sake of consistency across MCA and LCA participants. Scores on the RRB domain of the ADI were used to describe RRBs. These six questions assess participants’ circumscribed interests, adherence to routines or rituals, stereotyped or repetitive motor mannerisms, preoccupations with parts of objects, and sensory behaviors. In the current analyses, domain scores from the ADI (social interaction, nonverbal communication, and RRBs) were employed in analyses examining the relationship between autism features, aggression, and aggression change.

#### Adaptive Skills

##### Vineland Adaptive Behavior Scales (VABS)

The VABS is a semi-structured survey interview administered to primary caregivers (Sparrow et al., 2005). Trained clinicians ask caregivers open-ended questions about a child’s personal and social daily living skills. Answers are coded according to a 3-point Likert rating (0 = never; 1 = sometimes; 2 = usually) based on the participant’s typical independent completion of specified tasks. Adaptive behavior composite (ABC) scores are standard scores generated based three domains: communication skills, daily living skills, and socialization skills. Composite (ABC) scores from the VABS were employed in analyses examining the relationship between adaptive skills and aggression**.**

#### Intellectual Functioning

A hierarchy of standardized intelligence quotient (IQ) assessments were administered during face-to-face assessments using the most developmentally appropriate measure, based on language level and skill levels. Measures included the Wechsler abbreviated scale of intelligence (WASI; Wechsler, 1999), Wechsler intelligence scale for children (WISC-III; Wechsler, 1991), differential abilities scale (DAS; Elliott et al., 1990, 2007) and the Mullen scales of early learning (Mullen, 1995). When raw scores fell outside normative data ranges for standard scores, ratio IQs were calculated from age equivalents. In the current analyses, IQ standard scores from age 2 and age 9 were employed to examine the relationship between cognitive ability and both cross-sectional and longitudinal aggression.

### Data Analytic Plan

#### Preliminary Analyses

Each variable was carefully inspected for normality and outliers using histograms and box plots generated from SPSS; multiple variables were not normally distributed, and thus non-parametric tests were used as indicated, described below. Preliminary analyses were first conducted to determine associations between individual measures of aggression (ADOS, ADI, and CBCL) at each time point (ages 2, 9 and 18 for ADOS and ADI, and 9, 18, and 25 for the CBCL). Then, correlations were conducted between consecutive time points for all measures of aggression to determine consistency or variation in the occurrence and severity of participants’ aggression over time. For analyses involving ADOS-Aggression and ADI-Aggression, nonparametric correlations, specifically Kendall’s tau-b, were used due to the ordinal nature of the current data and violation of the assumption of normality. Finally, chi-square analyses were performed in order to determine whether there were significant differences in aggression (no aggression vs. some evidence of aggression) by autism diagnosis, recruitment site, and other demographic characteristics including sex, maternal education, and race.

#### Aim 1: Rates of Aggression over Time

To characterize rates of aggression in our sample, descriptive analyses were conducted using the Aggression Composite as well as all binary aggression variables at each time point. McNemar Tests were conducted in order to determine whether the proportion of participants who did and did not exhibit aggression changed significantly over time. The primary focus of analyses was the Aggression Composite, but for the sake of comprehensiveness, we also provide details regarding patterns for each aggression measure separately in instances in which these patterns deviated from the Aggression Composite score. Prevalence rates of aggression were examined both across the entire dataset as well as separately for MCA versus LCA individuals.

#### Aim 2: Longitudinal Patterns of Aggression

In order to characterize longitudinal patterns of aggression across all three time points, distinct Longitudinal Aggression profiles were created building on the work of Laverty and colleagues (2023). A total of nine longitudinal aggression profiles were possible (see Table 3). The Persistent profile was comprised of participants who displayed aggression on at least one of the three aggression measures (i.e., the aggression composite) at all time points. The absent profile was comprised of participants who did not display aggression on any measure at any time point. The decreasing and Increasing Profiles characterize participants whose aggression attenuated or initiated overtime, respectively (“Early” profiles indicate aggression decreased or increased by age 9, and “Late” profiles indicate that aggression decreased or increased by age 18). Finally, the Transient profile characterized participants who displayed aggression on at least one of the three aggression measures only during the second time point. Profiles with small sample sizes (i.e., below 10) were combined with the profile with the most similar pattern (e.g., early increasing and late increasing were combined to create the overall increasing profile). Final profiles included participants with at least two data points that were consistent with the primary pattern for that profile (e.g., the “absent longitudinal aggression profile” includes participants who did not display evidence of aggression at any time point with available data and had at least two available time points). Of note, CBCL-Aggression profiles were based on patterns across only two time points due to availability of data.

**Table 3:** Longitudinal aggression profiles and proportions

Measure (ages)						
	Ages 2 to 9 to 18				Age 9 to 18	Age 18 to 25
Profile	Aggression composite N (%)	ADOS-aggression N (%)	ADI-aggression-F N (%)	ADI-aggression-O N (%)	CBCL-aggression N (%)	CBCL-aggression N (%)
Persistent	56 (31%)	16 (9%)	23 (13%)	17 (10%)	0 (0%)	0 (0%)
Absent	24 (13%)	74 (42%)	56 (33%)	65 (38%)	20 (76%)	38 (81%)
Decreasing						
Late decreasing	17 (10%)	13 (7%)	27 (17%)	28 (18%)	3 (12%)	9 (19%)
Early decreasing	22 (13%)	37 (20%)	0 (0%)	0 (0%)		
Increasing						
Late increasing	44 (25%)	22 (12%)	13 (8%)	12 (7%)	3 (12%)	0 (0%)
Early increasing			32 (19%)	28 (17%)		
Transient	15 (8%)	14 (10%)	17 (10%)	17 (10%)	0 (0%)	0 (0%)

*ADOS* autism diagnostic observation schedule, *ADI-R* autism diagnostic interview revised, *TANT* tantrums, *F* aggression towards family, *O* aggression towards others, *CBCL* child behavior checklist

#### Aim 3: Factors Related to Aggression

Correlations of data from within each time point were first conducted in order to examine relations between aggression and participant factors including IQ (both verbal; VIQ and nonverbal; NVIQ), social components of autism symptoms (ADOS CSS SA and ADI total scores for social interaction and nonverbal communication domains), RRB components of autism symptoms (ADOS CSS RRB, and ADI total score for the RRB Domain), autism diagnosis, and adaptive behaviors (VABS adaptive behavior composite). Then, to determine whether participants with various Longitudinal Aggression profiles (identified in Aim 2) differed on these factors, as well as demographic characteristics (i.e., autism status, race, maternal education, sex, ethnicity), one-way ANOVAs were conducted. Significant *F* tests were followed up with pairwise comparisons using Bonferroni’s post-hoc correction to account for multiple tests.

#### Aim 4: Early Aggression Predicting Later Aggression

Finally, logistic regression analyses were performed to determine whether levels of aggression at age 2 or 9 predicted aggression later on (e.g., at ages 9 and 18), controlling for autism symptom severity (i.e., ADOS CSS), and cognitive functioning (e.g., NVIQ). Prior studies have found these factors to relate to aggression in autistic samples (Dominick et al., 2007; Hartley et al., 2008; Mazurek et al., 2013). Aggression Composite scores at ages 2 or 9 were employed as predictors in all logistic regression models in keeping with the current emphasis on multimethod measurement. Outcomes included the aggression composite as well as binary scores on ADOS-aggression, ADI-aggression, and CBCL-aggression during school-age and emerging adulthood.

## Results

### Preliminary Analyses

#### Correlations Between Aggression Measures and Across Time

Table 2 displays descriptives (sample size, mean/median, and range) and correlations among all aggression measures. The ADOS-aggression and ADI-aggression item(s) towards both family (ADI-aggression-F) and others (ADI-aggression-O) were largely uncorrelated, with the exception of weak positive associations at age 9 (ADOS-aggression and ADI-aggression-F; *τ*_b_ = 0.21,* p* = 0.005; ADOS-aggression and ADI-aggression-O: *τ*_b_ = 0.17,* p* = 0.03; *d* = 0.62–0.69). Unsurprisingly, the strongest correlations (large effect sizes) were found between ADI-aggression-F and ADI-aggression-O at both time points (age 9: *τ*_b_ = 0.65,* p* < 0.001; age 18: *τ*_b_ = 0.63,* p* < 0.001; *d* = 3.15–3.20). With respect to CBCL-aggression, positive correlations were found with both the ADI-aggression items across latter time points (CBCL-aggression with ADI-aggression-F, age 9: *τ*_b_ = 0.44,* p* < 0.001; age 18: *τ*_b_ = 0.39,* p* < 0.001 and CBCL-aggression with ADI-aggression-O, age 9: *τ*_b_ = 0.32,* p* < 0.001; age 18: *τ*_b_ = 0.46,* p* < 0.001; *d* = 1.1–1.7), but not with the ADOS.

Examining correlations across consecutive time points (e.g., from age 2–9, then 9–18, etc.) within each aggression measure, results revealed that associations across individual measures from age 2 to 9 to age 18 were not significant. Conversely, positive correlations (with large effect sizes; *d* = 0.94–1.3) were found from time points 9 to18 for both the ADI-Aggression-F and ADI-Aggression-O (*τ*_b_ = 0.28,* p* < 0.001; *τ*_b_ = 0.30,* p* < 0.001, respectively) and time points 18–25 and 9–25 for CBCL-Aggression (*r* = 0.54, *p* < 0.001; *r* = 0.49, *p* < 0.001, respectively). See Table 2 for further information.

#### Differences in Aggression by Autism Diagnosis and Demographic Variables

No significant differences were found in aggression on the basis of participants’ autism diagnosis, sex, maternal education, or race, according to the Aggression Composite or individual measures of aggression across all ages. Given that no differences in aggression were detected based on autism diagnosis, we feel even more confident combining the ASD and DD samples in subsequent analyses. However, significant differences were found in participants’ aggression according to recruitment site, such that at age 2 and 18, participants from Illinois were more likely (age 2: χ^2^ = 8.46, *p* = 0.004; age 18: χ^2^ = 6.78, *p* = 0.034) to exhibit definite aggression relative to the other sites (North Carolina and Michigan).

### Aim 1: Characterizing Rates of Aggression over Time

Overall, according to the Aggression Composite, approximately half (54.0%) of the current sample engaged in some form of aggression at age 2. At age 9, this proportion increased significantly, with 68.8% engaging in aggression (χ^2^ = 7.48, *p* = 0.006). A significant decline was evident between ages 9 and 18 (χ^2^ = 16.49, *p* < 0.001), with 41.8% of the sample presenting with aggression at age 18. Overall, individual measures of aggression (ADOS-Aggression, ADI-Aggression-O, CBCL-Aggression) followed a similar declining pattern across ages, with the exception of ADI-Aggression-F, which showed relatively consistent rates of aggression across time. Notably, at age 2, prevalence rates of aggression from clinician-rated observations (ADOS-Aggression) were higher relative to what was reported by parents in the ADI. Conversely, at ages 9 and 18, a higher proportion of the sample was noted to demonstrate aggression based on ADI-Aggression-F (age 9: 31.1%; age 18: 31.8%) and ADI-Aggression-O (age 9: 43.8%; age 18: 25.8%) compared to the ADOS (age 9: 29.7%; age 18: 9.1%). Prevalence rates were generally lowest according to the questionnaire measure (CBCL-Aggression age 9: 9.6%; age 18: 4.8%; age 25: 1.6%).

Patterns of aggression prevalence differed for MCA and LCA participants, such that rates were higher in LCA relative to MCA participants during school age and emerging adulthood according to the aggression composite as well as most individual measures of aggression. At age 2, according to aggression composite scores, LCA individuals (51%) and MCA participants (61%) tended to display relatively similar rates of aggression. In contrast, at age 9, the discrepancy between LCA (80%) and MCA (48%) individuals’ aggression reaches 32%. This discrepancy becomes even more pronounced at age 18, with around 36% more LCA (58%) participants exhibiting aggression compared to MCA (22%) individuals.

### Aim 2: Longitudinal Patterns of Aggression

Various profiles of aggression over time—referred to as longitudinal aggression profiles—were identified based on participants’ patterns of aggression across ages 2, 9, and 18. Based on the Aggression Composite, the largest proportion of the sample (approximately 31%) was characterized by persistent aggression at all time points. The next largest profile consisted of approximately a quarter of the sample (25%) and was characterized by no observed or reported aggression in early childhood with aggression emerging later in life (either at age 9 or 18); we refer to this profile as Increasing. The Decreasing profiles, characterized by initial evidence of aggression followed by its later absence, also comprised nearly a quarter of the sample (23% overall; 13% early decreasing and 10% late decreasing). Next, the absent profile, characterized by no evidence of aggression across all available time points, comprised 13% of the sample. The smallest proportion of the current sample (~ 8%) was characterized by Transient aggression, reflective of individuals who displayed aggression *only* at the second time point, but not the first or third. Notably, this profile was limited to participants with complete data at all three time points. See Table 3 for further information.

A few notable differences exist in sample sizes of longitudinal aggression profiles that were created based on individual measures of aggression, as opposed to the Aggression Composite. Similar proportions of the sample make up the Transient, Decreasing, and Increasing profiles across all metrics of aggression used in the current study. In contrast, more marked differences exist between the Aggression Composite and individual measures of aggression in terms of the proportion of participants in the Absent versus Persistent profiles. Approximately a third of the sample displayed persistent aggression according to the Aggression Composite, while this value dropped to 0–13% based on individual aggression measures.

### Aim 3: Factors Related to Aggression

#### Verbal and Nonverbal IQ

Significant negative correlations were found between IQs (verbal and nonverbal) and Aggression Composites at age 9 and 18. Those higher in either VIQ and NVIQ were less likely to have evidence of aggression at both age 9 and 18, but not at age 2. Similar patterns were found for all separate aggression measures, except CBCL-Aggression at age 9 (Table 4).

**Table 4:** Relationships between individual characteristics and the aggression composite within time point and longitudinally

	Descriptive statistics				Within time point correlations			Comparisons between longitudinal aggression profiles						
Age 9 individual characteristic	Mean	SD	Min	Max	Age 2	Age 9	Age 18	Group 1-absent	Group 2-late decreasing	Group 3-early decreasing	Group 4-persistent	Group 5-increasing	Group 6-transient	F (df)
IQ-verbal	53.9	37.7	3	141	0.05	** − 0.33**	− 0.20*	38.3	47.9	50.3	35.9	35.0	34.0	2.21
IQ-nonverbal	63.2	33.4	2	151	0.10	** − 0.29**	** − 0.26**	**68.9**	**74.5**	**83.1**	**66.9**	**60.6**	**62.8**	**4.12**^**a,c,d**^
ADOS-SA CSS	11.2	5.9	0	20	0.07	**0.34**	0.04	6.75	7.29	5.95	7.71	7	7.85	1.36
ADI (A)	20.9	8.3	0	30	0.15*	**0.20**	0.13	13.0	16.9	14.2	17.59	15.7	15.6	2.13
ADI (BNV)	9.33	4.33	0	14	0.01	**0.18**	0.10	**9.25**	**10.9**	**8.3**	**10.6**	**10.7**	**11.4**	**2.58**
ADOS-RRB CSS	3.83	2.51	0	8	0.14*	**0.21**	**0.23**	**4.92**	**5.65**	**5.1**	**6.7**	**5.61**	**6**	**2.46**^**b**^
ADI (C)	5.29	2.93	0	12	**0.15**	**0.17**	**0.21**	**2.25**	**2.94**	**3.26**	**3.84**	**3.39**	**3.47**	**2.57**^**b**^
Vineland ABC	51.4	26.8	17	117	0.02	** − 0.29**	** − 0.37**	65.43	63.4	64.4	61.68	60.8	59.9	1.54

Bold values indicate significant differences at the .05 level

Significant pairwise comparisons with Bonferroni correction: a = 3 versus 5; b = 1 versus 4; c = 3 versus 6; d = 3 versus 4

*ADOS* autism diagnostic observation schedule, *ADOS-SA CSS* ADOS social affect calibrated symptom severity, *ADOS-RRB CSS* ADOS repetitive and restricted behaviors calibrated symptom severity, *ADI-R* autism diagnostic interview revised, *ADI-R (A)* ADI-R social interaction domain, *ADI-R (BNV)* ADI-R nonverbal communication domain, *ADI-R (C)* ADI-R repetitive and restricted behaviors domain, *Vineland ABC* adaptive behavior composite score on VABS

*Significant differences at the .01 level

Similarly, NVIQ differed significantly across aggression composite longitudinal profiles *F*(5) = 4.12, *p* = 0.001; (ƞ^2^ = 0.1) with a large effect. A large effect size emerged for the ADOS-Aggression (ƞ^2^ = 0.11), and a moderate effect emerged for ADI-Aggression-F (ƞ^2^ = 0.07). Post-hoc analyses revealed that participants in the Early Decreasing group had significantly higher NVIQs (*M* = 83.1; *SD* = 18.4) than those in the Persistent (*M* = 35.9, *SD* = 21.5), Increasing (*M* = 35.0, *SD* = 21.4), or Transient (*M* = 34.0, *SD* = 21.9) profiles according to the Aggression Composite. Similar patterns were observed based on individual aggression measures such that the Decreasing profile for ADOS-Aggression (Early Decreasing) and ADI-Aggression-F (Late Decreasing) had higher NVIQs (ADOS-Aggression: *M* = 78.5; *SD* = 18.2; ADI-Aggression-F: *M* = 79.1; *SD* = 20.2) than those with other profiles (Increasing and Transient for ADOS-Aggression; Persistent for ADI-Aggression-F).

#### Autism Symptoms—Social Communication

Significant positive correlations were found between the Aggression Composite and ADOS CSS-SA (age 9; *d* = 1.18), and ADI Social Interaction (ages 2 and 9; *d* = 0.48; *d* = 0.65, respectively) and Communication domains (age 9; *d* = 0.58). Those with less severe social communication symptoms were found to exhibit less aggression in toddlerhood and school-age. Similar associations were found for all individual aggression measures, with the exception of the CBCL, which was unrelated to social and communication symptoms on both the ADOS and ADI. ADOS-Aggression also showed additional associations at age 18 with Social and Communication symptoms on the ADOS and ADI. See Table 4 for correlation coefficients.

No differences in ADOS-CSS-SA were found between overall Aggression Composite Longitudinal Profiles. In contrast, significant differences in ADOS-CSS-SA were detected between Longitudinal Aggression profiles based on ADOS-Aggression [*F*(5) = 2.78, *p* = 0.02)] and ADI-Aggression-O [*F*(5) = 2.24, *p* = 0.05)]. Moderate effect sizes emerged for ADOS-Aggression (ƞ^2^ = 0.08) and ADI-Aggression-O (ƞ^2^ = 0.07). Post-hoc analyses revealed that based on the ADI-Aggression-F, the Absent profile had a lower average ADOS CSS SA (*M* = 6.54; *SD* = 2.96) than the Transient profile (*M* = 9.00; *SD* = 1.50). Post-hoc analyses for other ADOS Longitudinal Aggression profiles did not reveal significant differences.

ADI communication domain scores in the nonverbal communication subdomain differed significantly between aggression composite longitudinal profiles [*F*(5) = 2.58, *p* = 0.028; ƞ^2^ = 0.07] and for ADOS-Aggression [*F*(5) = 2.25, *p* = 0.05; ƞ^2^ = 0.06] only. Effect sizes were moderate, however no post-hoc pairwise comparisons were significant.

Although there were no significant differences in ADI social interaction domain scores across aggression composite longitudinal profiles, significant differences were found in ADI Social Interaction Domain Scores between longitudinal profiles based on ADOS-Aggression as well as ADI-Aggression-O. Moderate effect sizes were detected for ADOS-Aggression (ƞ^2^ = 0.08) and ADI-Aggression-O (ƞ^2^ = 0.07). Post-hoc analyses revealed that the Absent profiles(s) had significantly lower ADI Social Interaction Domain scores (ADOS-Aggression: *M* = 14.6; *SD* = 6.56; ADI-Aggression-O: *M* = 13.2; *SD* = 6.38) than the Increasing (ADOS-Aggression: *M* = 19.2; *SD* = 5.84), Persistent (ADI-Aggression-O: *M* = 20.2; *SD* = 6.24), and Transient (ADI-Aggression-O: *M* = 19.2; *SD* = 4.83) aggression profiles.

#### Autism Symptoms—RRBs

Significant positive correlations with moderate to large effect sizes were found between the Aggression Composite and RRB scores on the ADOS (ages 2, 9, and 18; *d* = 0.44;* d* = 0.69;.*d* = 0.76, respectively) and the ADI (ages 2, 9, and 18; *d* = 0.48;* d* = 0.55;.*d* = 0.69, respectively); those with fewer RRBs were less likely to demonstrate aggression. Similar patterns emerged for individual aggression measures, except for CBCL-Aggression which was uncorrelated with all RRB metrics.

ADOS RRB scores were found to differ significantly across aggression composite longitudinal profiles [*F*(5) = 2.46, *p* = 0.03, ƞ^2^ = 0.07]. More specifically, in post-hoc tests, the absent profile had significantly lower RRBs (*M* = 4.92; *SD* = 2.41) relative to the Persistent profile (*M* = 6.70; *SD* = 2.24). Individual measures of aggression including the ADOS-Aggression, ADI-Aggression-O showed similar patterns to the Aggression Composite Longitudinal Profiles.

ADI RRB Domain scores were found to be significantly different across aggression composite longitudinal profiles [*F*(5) = 2.57, *p* = 0.03; ƞ^2^ = 0.07] with a moderate effect size. Post-hoc analyses revealed that the Persistent profile had significantly higher ADI RRB Domain scores (*M* = 3.84; *SD* = 1.77) than the absent longitudinal aggression profile (*M* = 2.25; *SD* = 1.54). No significant differences in RRB symptoms reported in the ADI were found for other groups or for Longitudinal Aggression profiles based on each individual aggression measure.

#### Adaptive Behavior

Significant negative correlations were found between the VABS ABC and all individual aggression measures at ages 9 and 18 (except CBCL-Aggression, which had significant negative correlations only at age 18); those with better adaptive functioning were less likely to exhibit aggression.

The VABS ABC did not differ across aggression composite longitudinal profiles (Table 4). Although significant differences in the VABS ABC were found between ADOS-aggression longitudinal profiles [*F*(5) = 2.78, *p* = 0.02; ƞ^2^ = 0.07], no post-hoc comparisons were significant.

#### Demographic Characteristics

Finally, participant demographics (e.g., autism status, race, maternal education, sex, and ethnicity) were compared across Longitudinal Aggression profiles, with no differences on any of the factors (Table 5).

**Table 5:** Demographic characteristics and frequencies of participants according to longitudinal aggression profile

	Group 1-absent	Group 2-late decreasing	Group 3-early decreasing	Group 4-persistent	Group 5-increasing	Group 6-transient	Chi-squared	p
Race							3.94	0.56
White/Caucasian	16	15	14	44	28	11		
African American	8	6	9	12	16	4		
Ethnicity							3.78	0.58
Hispanic	23	20	22	53	44	14		
Not hispanic	0	1	1	3	0	1		
Maternal education							8.83	0.12
< 4 years of higher education	14	4	13	27	23	5		
> 4 years of higher education	10	14	10	22	18	9		
Sex							4.16	0.53
Male	20	14	19	44	38	11		
Female	4	7	4	12	6	4		
Autism status							3.08	0.69
ASD	21	17	18	49	35	14		
DD	3	4	5	7	9	1		

### Aim 4: Early Aggression Predicting Later Aggression

Logistic regressions with early aggression (at 2 or 9) as predictors for later aggression (at age 9 or 18) were conducted, employing Benjamini–Hochberg corrections for multiple tests. Results suggest that aggression at age 2 was not a significant predictor of aggression at ages 9 or 18, when controlling for NVIQ and CSS. However, aggression at age 9 significantly predicted aggression at 18 [Exp(B) = 3.72; 95% CI [1.28, 10.82]; *p* = 0.036] above and beyond the effects of NVIQ and CSS. Odds ratios revealed that participants who displayed any evidence of aggression at age 9 were 3.72 times likelier to exhibit aggression at 18, accounting for differences in autism symptoms and nonverbal cognitive ability. This is considered a medium effect (Chen et al., 2010). Composite aggression at age 9 also significantly predicted aggression towards family at age 18 [ADI-Aggression-F; Exp(B) = 3.56; 95% CI [1.31, 9.46]; *p* = 0.036], however this finding was marginal when controlling for NVIQ and CSS [ADI-Aggression-F; Exp(B) = 3.09; 95% CI [1.12, 8.76]; *p* = 0.072].

## Discussion

The current study examined aggression in autism and other NDDs using multimethod measurement and longitudinal methodology. Our findings replicate previous research that identified high rates of aggression in these populations (Kanne & Mazurek, 2010; Lee et al., 2006; Mazurek et al., 2013), and extends this work by following the same participants from early childhood into emerging adulthood. Rates of aggression (e.g., tantrums, hitting, biting, using implements) ranged from just over half the sample during childhood to approximately a third of the sample in emerging adulthood, with a variety of patterns of change in-between. Notably, LCA participants were reported to have greater rates (around 18% more on average) of aggression compared to MCA participants. Consistent with previous work, individual characteristics (e.g., lower NVIQ, more RRBs) not only increased the likelihood of concurrent aggression, but also related to patterns of aggression over time. Although aggression in toddlerhood did not predict later aggression, aggression during school-age strongly predicted aggression in early adulthood. These insights highlight particular risk factors (e.g., low NVIQ) and important developmental periods (e.g., school-age) that may shape patterns of aggression over time in this population that merit our attention in assessment and treatment.

### Rates of Aggression over Time

The current work examined rates of aggression throughout toddlerhood, school-aged, and emerging adulthood among people with autism or NDDs. Although a substantial proportion of our sample demonstrated aggression at each time point (toddlerhood: 54%; school-age: ~ 69%; and emerging adulthood: ~ 42%), it was encouraging to see rates of aggression decline with age. Over half of the current sample displayed aggression at age 2 according to observational and parent-interview methods. Just over two-thirds of the sample was found to engage in some form of aggression around age 9, with a 27% decrease in aggression by age 18. As reported by other authors, rates of aggression are particularly high in autism and NDD populations (Broidy et al., 2003; Hartley et al., 2008; Laverty et al., 2023). These findings also align with prior cross-sectional studies, which found a negative relationship between age and likelihood of aggression. (Hartley et al., 2008; Kanne & Mazurek, 2010; Matson & Rivet, 2007).

Although our overall pattern of findings is consistent with prior work, it is important to note that the estimated rate of aggression at age 2 in our sample (54%) was much larger than what has been found by previous studies (e.g., 22.5% for a sample of autistic children with a mean age of 3.5 years and comparable cognitive ability to the current sample; Hartley et al., 2008). This inconsistency may relate to specific metrics of aggression used in the current study, which includes reports and observations of tantrums as the age 2 proxy for aggression. A previous study, for example, used the CBCL aggression subscale, which covers, and perhaps sets a higher bar for different forms of aggression. Although tantrums have been used as a proxy for more direct aggression and conceptualized as a precursor to aggression (Hay et al., 2014), tantrums in toddlers are likely more common than other more severe forms of aggression.

Consistent with the notion that metrics of aggression may influence estimates, the current analyses found rates of aggression to vary across measures, not surprisingly, with substantially lower estimates based on ADOS-Aggression compared to the other metrics (e.g., ADI) and the Aggression Composite. Methodological considerations are critical for interpreting this data. Because the ADOS is an observational measure, the window of opportunity to observe aggression is narrower compared to parent interviews and questionnaires, which provide a more comprehensive depiction of behavior across time and contexts. The laboratory setting of the ADOS with an unfamiliar examiner may also influence aggression—children may be more likely to display aggression in familiar environments with familiar people (De Giacomo et al., 2016). Nonetheless, the ADOS aggression measure in the current study captured aggression in some participants whose behaviors may not have been reported by parent report or interview alone, emphasizing the need to use observational methods in tandem with other modalities when measuring aggression.

Given the particularly high rates of aggression at age 9, research on difficulties in school-age may also prove relevant to the current findings. Increasingly complex peer issues and social challenges in a school environment likely impact behavioral challenges during school age (Horiuchi et al., 2014). Results also suggest the most marked difference in aggression rates between MCA (48%) and LCA (80%) participants occurred at age 9, which supports the idea that school-age may be an especially difficult time for less-cognitively-abled autistic people as they navigate a variety of school-related challenges, both academically and behaviorally.

In contrast, later adolescence/emerging adulthood had the lowest rates of aggression. Indeed, a general downward trend was observed in aggression in both MCA and LCA subgroups. Various developmental studies have shown that capacity for self-regulation and inhibitory control increase with age (van den Bergh et al., 2014) in autism. Furthermore, declines in aggression over time may also be due in part to interventions and supports received to promote social, adaptive, or emotional skills, although this was not empirically tested in the current study. These results add to findings that indicate general longitudinal declines in aspects of behaviors used to define autism (e.g., severity and frequency of RRBs) as well as symptoms of co-occurring psychiatric conditions (Georgiades et al., 2022; Laverty et al., 2023; Magiati et al., 2014; Simonoff et al., 2020; Woodman et al., 2015).

### Longitudinal Patterns of Aggression

Analyses from the current study indicate that patterns of change in aggression vary over time among different autistic people and those with other NDDs –findings that can only be revealed using longitudinal data. While the greatest proportion of the current sample (approximately a third), according to Aggression Composite scores, was characterized by aggression at all available time points, half of the sample included people who either improved (approximately a fourth) or worsened (approximately a fourth) in their aggression over time. These findings may be of interest not only to clinicians and researchers, but also to families of autistic toddlers with high rates of tantrum behaviors. It is nearly equally likely for aggression to improve as it is to persist, emphasizing the need for further longitudinal investigation and tailored interventions.

Relative sample sizes for the Longitudinal Aggression profiles in the current sample did not corroborate Laverty and colleagues’ (2023) findings. Their aggression profile ranks (from most to least participants) were: Absent, Decreasing, Persistent, and Increasing, while the current results suggested the following order: persistent, increasing, decreasing, absent—a nearly opposite pattern. Our study had more people who developed aggression over time or showed persistent aggression over time than found by the previous research team. These differences could be due to several factors including differences in the time points used (i.e., 12–24 years for Laverty et al. vs. 2–25 for the current work), and our use of multiple metrics of aggression to form the Aggression Composite (as opposed to a single questionnaire). Our methods allowed for more opportunities to elicit reports and observations of aggression, likely leading to the larger Persistent and Increasing profiles. This contrast further supports the need for future multi-method assessment of aggression in autism and other NDDs.

### Factors Related to Aggression

Correlational findings in the current study were consistent with prior cross-sectional research investigating factors related to aggression (Hartley et al., 2008; Kanne & Mazurek, 2010; Matson & Rivet, 2007; Murphy et al., 2005). Specifically, VIQ and NVIQ, autism symptoms, and adaptive behavior were correlated with aggression in the expected directions at most time points. Aggression was negatively correlated with adaptive skills and IQ, and positively correlated with ASD symptomatology.

Out of all the individual characteristics examined in this study, NVIQ and RRB symptoms were most related to aggression change over time in addition to within time points. While some other significant associations were within one measure only (and may be influenced by shared methods variance), we are encouraged by the cross-measure longitudinal findings, which bolster our findings in these analyses. The current study found expected social correlates with aggression within most time points (e.g., ADOS CSS SA, ADI Social Interaction Domain score), though these social components did not relate to changes in aggression over time, suggesting that social communication challenges relate to concurrent aggression but do not relate as much to trajectories of aggression found by the Aggression Composite. Poor social communication in individuals of a particular age (e.g., school age) may lead to frustration and ultimately to instances of aggression when other means of problem-solving do not suffice. While these social communication differences do not consistently manifest over time, they can impact how a child’s social communication skills are perceived. This aligns with previous research which recognizes clear functional differences between repetitive behaviors and social aspects of the autism phenotype (Lord et al., 2001).

Notably, only NVIQ was different between the decreasing and persistent longitudinal aggression profiles, which is consistent with prior research suggesting nonverbal cognitive functioning as most strongly linked to higher aggression (relative to verbal cognitive functioning scores on the Mullen scales of early learning; Hartley et al., 2008). For nonverbal or minimally verbal individuals, aggression may communicate needs that otherwise go unmet. Interventions that promote useful communication, such as Augmented and Alternative Communication or functional communication training, could help reduce persistent aggression (Ghaemmaghami et al., 2021; Hartley et al., 2008; Lindgren et al., 2020).

RRB symptoms (coded in both the ADOS and ADI) were highest for those in the Persistent aggression profiles. These findings support work examining the relationship between more broadly defined externalizing behaviors in autism and RRBs (Dominick et al., 2007). Brown and colleagues (2019) suggest that RRBs may be an alternative to aggression by potentially self-soothing, and other research teams have suggested that RRBs may play a role in stress reduction or escape from difficult situations (Lewis & Bodfish, 1998), or may occur due to being more or equally as enjoyable as other social interactions (e.g., Klintwall & Eikseth, 2012). Ultimately, RRBs present heterogeneously, and given that those with persistent aggression and high levels of RRBs also generally have lower adaptive, social, and leisure skills (Leekam et al., 2011), it is also possible that aggression and RRBs occur because of these underlying challenges.

Finally, autistic vs NDD participants did not differ in their level of aggression or how their aggression changed over time. This is consistent with prior studies that found more similarities than differences between autistic and NDD groups in this sample (Schiltz et al., 2023) and others (Chan et al., 2018; Hazlett et al., 2009).

### Early Aggression Predicting Later Aggression

Prior work on the relationship between early and later aggression in autism is limited. Some research has found that more severe challenging behaviors (like aggression) may be observed when less severe behaviors (like tantrums) do not function for the child in terms of getting needs met (e.g., Warner et al., 2020). The current results suggest that the presence of aggression in toddlerhood does not provide meaningful information regarding aggression at 9 or 18 years old. Instead, participants were nearly four times likelier to engage in aggression at 18 if aggression was displayed at age 9. As mentioned above, because the current method employed tantrum data from age 2, it may be the case that such proxy behaviors are less clearly related to later experiences of aggression relative to the consistent metrics of aggression used at both 9 and 18. Although such proxy behaviors like tantrums better capture families’ and participants’ experiences with earlier experiences of challenging behavior, these behaviors did not predict later development of aggression.

## Implications

The current findings have implications for both providers and families. Based on high rates of aggression and value of aggression at school-age in predicting difficulties in emerging adulthood, the school-age years seem to be an especially important time in autistic children’s behavioral development. This was particularly the case for children with lower NVIQs (approximately 60–65), or who had high RRBs (see Brown et al., 2019). Notably, rates of aggression towards family remained relatively stable over time; therefore, families may benefit from additional behavioral supports and parent coaching programs across their child’s development. Early intervention may also be especially important to consider, as learning to replace aggression with other skills during early development may prevent aggression in later years (Brosnon & Healy, 2011). We also recommend that clinicians be especially vigilant in prioritizing observations and reports of aggression in their school-age patients, with the idea that aggression detected and treated early on in these years could potentially prevent issues over time. Indeed, even brief references to aggression noted in an ADOS, ADI, or CBCL may be worth following up.

## Limitations and Future Directions

Despite the strengths of the current work, there are limitations. First, although the current sample was large overall, sample sizes pertaining to analyses with the CBCL, as well as examinations of aggression change across consecutive time points, are smaller, which means they should be interpreted cautiously and replicated with larger samples. Generalizability of findings from the current work may also be limited due to the specificity of the cohort and ages of participants used in the current study. That is, the generalizability of the current results to other clinical samples (e.g., participants diagnosed later in development or after the 1990s) remains unclear. Advances in autism intervention since the 1990s and early 2000s when the current participants were school age may mean that children today could have different behavioral outcomes and experiences than those in the present study. Treatments for aggression are now increasingly available (e.g., Machalicek et al., 2007). Second, the current analyses were unable to control for unequal attrition of participants by race and maternal education, which should be addressed in future studies. Although participants in the current study reflected a wide range of developmental stages (e.g., toddlerhood, emerging adolescence, and emerging adulthood), middle and older adulthood were not included. Future work should replicate these findings using other clinical samples that are followed later into adulthood.

Although this study incorporated multimethod measurement to represent aggression as holistically as possible, it should be noted that the ADOS as a metric of aggression may capture aggression differently than other more commonly used measures. Nevertheless, the ADOS E codes (e.g., the E2 code used for the present analyses) have shown strong relationships with emotional-behavioral problems (EBPs) and have been suggested by prior research teams as appropriate metrics of externalizing behaviors like aggression (Galligan et al., 2021). However, correlations between aggression captured by the ADOS and other measures (e.g., the ADI) were weak, indicating a need for future work to continue investigating the relationship between aggression coded on the ADOS versus other measures.

More information is also needed about the various forms aggression may take (e.g., tantrums, physical striking out, disruptive use of objects, shouting, or verbal abuse) and towards whom it may be directed. Future researchers may be encouraged to investigate how the severity of participants’ challenging behavior changes over time (e.g., whether participants with certain profiles experience changes in the severity or number of behavior topographies they display), helping clinicians more precisely target their intervention efforts. In order not to reify individual measures of aggression, binary variables of definite versus no aggression were created in the current work, instead of metrics of frequency or severity. While these binary variables were well suited for the current analyses, further studies that carefully address these issues are needed (e.g., methodologically employing other clustering or classification approaches to derive aggression profiles, such as Growth Mixture Modeling), as insight into the nuanced nature of aggression remains limited.

## Conclusion

Among people with ASD or NDDs, aggression is prevalent, especially for LCA individuals. While rates peaked at around age 9 and declined in emerging adulthood for many people, this pattern was not universal. Emerging adults with aggression were almost four times likelier to have demonstrated aggression during school-age and were found to have high rates of RRBs throughout development. NVIQ was protective in some ways, such that even for those who displayed aggression as toddlers, higher NVIQ related to attenuation in the likelihood of aggression over time. Special attention should be paid to school-age periods, given the high rates of aggression at this age and strong prediction of later aggression in emerging adulthood. This study provides evidence that while aggression is common in autism and NDDs, certain developmental periods (school-age) and high-risk subgroups (low IQ, higher RRBs) warrant targeted supports.
